# Association of Serum Thyroid Profile and Glycated Hemoglobin Levels in Diabetic Patients: A Cross-Sectional Study

**DOI:** 10.7759/cureus.92213

**Published:** 2025-09-13

**Authors:** Jahnabi Baruah, Sonit Kalita, Pawan Kumar, Anand Gupta, Deepika Lahon, Julie Sarmah

**Affiliations:** 1 Biochemistry, Tezpur Medical College, Tezpur, IND; 2 Psychiatry, Nagaon Medical College, Nagaon, IND; 3 Biochemistry, Nalbari Medical College, Nalbari, IND; 4 Biochemistry, Dhubri Medical College, Dhubri, IND

**Keywords:** diabetic complications, diabetic dyslipidemia, glycosylated hemoglobin, hba1c, subclinical hypothyroidism, t3, t4, thyroid hormones, tsh

## Abstract

Introduction

Diabetes mellitus (DM) is a chronic metabolic disorder frequently associated with thyroid dysfunction and lipid abnormalities, which may worsen glycemic control and increase cardiovascular risk. The interplay between thyroid hormones and glucose metabolism is especially important in patients with type 2 diabetes mellitus (T2DM). This study was conducted to assess whether thyroid hormone levels (TSH, total T3, total T4) differ between diabetes and matched controls and whether they correlate with HbA1c within groups and comparison of lipid profiles between groups.

Methods

A cross-sectional study was conducted at Tezpur Medical College and Hospital, Assam, India, including 100 diabetic patients and 100 age- and sex-matched non-diabetic controls. Blood samples were analyzed for HbA1c, thyroid-stimulating hormone (TSH), total triiodothyronine (T3), total thyroxine (T4), and lipid profile using standard automated analyzers. Statistical analysis included Student’s t-test, Pearson correlation, and multiple linear regression.

Results

Diabetic patients had significantly higher levels of HbA1c, total cholesterol, triglycerides, low-density lipoprotein (LDL), and very-low-density lipoprotein (VLDL) compared to controls. Total T3 and total T4 levels were significantly lower in diabetics, while TSH levels showed a non-significant increasing trend. HbA1c correlated negatively with total T3 and total T4 and positively with lipid parameters. All statistical analyses were done with the help of Microsoft Excel and Jamovi software.

Conclusion

In this study, cases with diabetes had lower total T3 and T4 levels than healthy controls, but their TSH levels were the same. HbA1c exhibited only weak correlations with thyroid indices, suggesting an absence of a robust glycemic-thyroid association in this dataset. Routine screening for lipid abnormalities may help improve metabolic control and reduce complications in diabetic patients.

## Introduction

Diabetes mellitus is a long-term condition that disturbs the normal metabolism of carbohydrates, fats, and proteins. The disorder occurs due to insufficient insulin release, decreased tissue responsiveness to insulin, or a combination of these factors. It can result in both acute and chronic complications, contributing substantially to illness and premature mortality [1]. The disease affected 537 million individuals between the ages of 20 and 79 in 2021, according to estimates. The International Diabetes Federation (IDF) predicts that this number could increase to 643 million by 2030 and 783 million by 2045 [2].

The thyroid gland is also another vital endocrine organ and is essential for regulating metabolic processes, including the utilization of carbohydrates and the secretion of insulin. Research indicates that diabetes mellitus can alter thyroid function, while thyroid abnormalities can also influence blood sugar control. This reciprocal relation involves factors such as altered thyroid-stimulating hormone (TSH) release, changes in the conversion of total thyroxine (T4) to total triiodothyronine (T3) in peripheral tissues, and elevated insulin levels exerting direct effects on thyroid gland function [1].

Both diabetes mellitus and thyroid disorders are among the most common endocrine diseases worldwide. Abnormal thyroid function may adversely influence blood sugar regulation and accelerate the development of chronic diabetic complications [3]. These two conditions share pathophysiological connections, including autoimmune mechanisms, associations with metabolic syndrome, and effects on cellular metabolism. In hyperthyroidism, heightened production of glucose transport proteins in the liver has been observed, while the level of total T3 within cells can modify how sensitive tissues are to insulin by regulating GLUT4-mediated glucose uptake in skeletal muscle [4].

Type 2 diabetes mellitus (T2DM) and thyroid disease frequently present with overlapping clinical manifestations such as edema, fatigue, pallor, and weight changes, which may obscure the diagnosis of one condition in the presence of the other [4]. Insulin resistance in T2DM is often characterized by high blood sugar, elevated insulin concentrations, and altered iron metabolism, as reflected in increased serum ferritin levels. Excess iron in the body may promote diabetes through oxidative damage to pancreatic β-cells, impaired hepatic insulin clearance, and disruption of normal hepatic glucose regulation. Persistent hyperglycemia also leads to the glycation of proteins, including hemoglobin, and produces advanced glycation end products (AGEs). In diabetic individuals, high ferritin concentrations in the blood are linked to suboptimal control of blood glucose, as indicated by elevated glycated hemoglobin (HbA1c) values [5].

Dyslipidemia is one of the common metabolic abnormalities in diabetic patients, characterized by increased levels of different biomolecules like triglycerides, reduced high-density lipoprotein cholesterol (HDL-C), and the presence of small dense low-density lipoprotein (LDL) particles. This “atherogenic lipid triad” significantly concerns the risk of cardiovascular disease in diabetes. Thyroid dysfunction can further exacerbate lipid abnormalities - hypothyroidism is often associated with hypercholesterolemia and elevated LDL-C, whereas hyperthyroidism may lower total cholesterol but increase lipolysis and free fatty acid turnover. This triad of dyslipidemia, thyroid disorder, and poor glycemic control can create a synergistic effect, accelerating atherosclerosis and cardiovascular complications in diabetic individuals [6].

## Materials and methods

Study design

A case-control with cross-sectional sampling was conducted over six months; this study was carried out in the Department of Biochemistry, Tezpur Medical College and Hospital (TMCH). The study popula­tion consisted of 200 individuals - 100 diabetic patients serving as the case group and 100 healthy volunteers (hospital staff/attendants) without diabetes or thyroid disease, age (±5 years) and sex matched to cases serving as controls. For every participant, the following biochemical parameters were assessed: fasting blood glucose, postprandial blood glucose, blood urea, serum creatinine, fasting lipid pro­file, serum thyroid-stimulating hormone (TSH), total triiodothyronine (T3), total thyroxine (T4), and glycated hemoglobin (HbA1c).

Serum TSH, total T3, and total T4 were analyzed by chemiluminescent immunoassay on the Vitros 5600 autoanalyzer (Ortho Clinical Diagnostics, Raritan, NJ, USA), and HbA1c was measured using the high-performance liquid chromatography (HPLC) method on the Bio-Rad D-10 analyzer (Bio-Rad Laboratories, Hercules, CA, USA). The estimation of fasting and postprandial blood glucose, blood urea, serum creatinine, and lipid profile was performed on a semi-autoanalyzer (DiaSys Diagnostic Systems GmbH, Holzheim, Germany) in the Department of Biochemistry. Reliability was ensured by implementing daily internal quality control procedures, and instruments were calibrated as per the manufacturer’s guidelines. Furthermore, stan­dard operating procedures will be strictly followed to ensure both accuracy and precision.

Inclusion criteria consisted of diagnosed cases of type 1 or type 2 diabetes mellitus who were either attending or admitted to the Department of Medicine, TMCH. Type 1 diabetes was defined based on clinical presentation and insulin dependence from diagnosis, while type 2 diabetes was identified according to the recommendations of the American Diabetes Association (ADA) and the World Health Organization (WHO) 2025. Both male and female participants, irrespective of socioeconomic background, were included. The study included healthy volunteers (hospital staff/attendants) without diabetes or thyroid disease, who were age- and sex-matched to the cases, serving as controls.

Exclusion criteria comprised individuals with known thyroid disorders, hypertension, alcohol consumption, or active smoking. Patients currently on or with recent (within three months) use of iron supplements, those with hemoglobinopathies or glucose-6-phosphate dehydrogenase (G6PD) deficiency, and individuals with a history of aplastic anemia due to drugs such as chloramphenicol, phenylbutazone, or carbamazepine were excluded. Participants with anemia (hemoglobin <12 g/dL in women and <13 g/dL in men) or those recently treated for anemia, as well as pregnant women, were excluded. Individuals with chronic liver disease, kidney disorders, malignancy, acute infections, recent myocardial infarction, or bleeding disorders were also not considered. Furthermore, patients who did not provide informed consent were excluded from the study.

## Results

**Figure 1:**
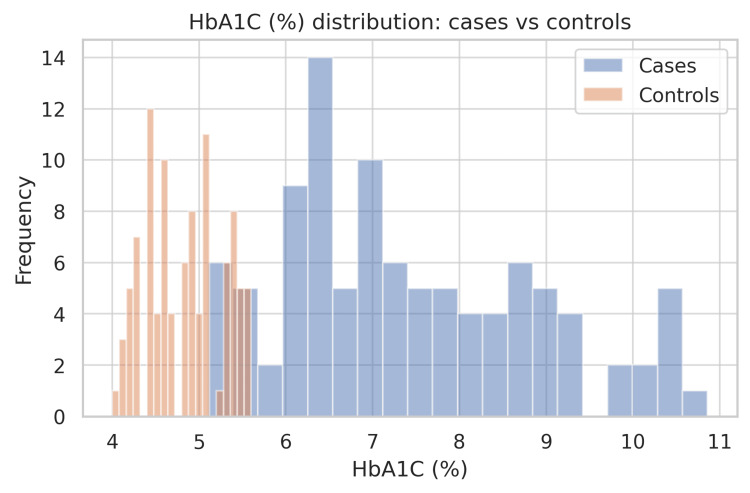
Frequency distribution of HbA1c in diabetic cases and healthy controls. The histogram displays the HbA1c values for 100 cases (blue) and 100 controls (orange). The distribution in cases is shifted to the right, indicating poorer glycemic control. Group means (±SD) were 7.42 ± 1.46% in cases and 4.83 ± 0.45% in controls, an absolute difference of +2.59%; the between-group comparison was statistically significant (p < 0.001). The standardized mean difference (Cohen’s d) was ~2.40, indicating a substantial effect size. Bins were chosen to display both groups on the same axis for direct visual comparison.

The current study included 200 participants, 100 of whom were diabetic cases and the remaining 100 were non-diabetic controls. Table 1 summarizes the baseline characteristics and biochemical parameters of both the diabetic and non-diabetic groups. The average age of the participants was very similar between the two groups (49.28 ± 10.59 years in cases vs. 49.58 ± 18.03 years in controls), and this difference was not statistically significant (p = 0.886). This shows that the groups were well-matched for age. The glycemic status shows a significant difference. HbA1c levels were much higher in the diabetic group compared to the controls (7.42 ± 1.46% vs. 4.83 ± 0.45%; p < 0.001). Diabetics had HbA1c values nearly 2.6% higher than non-diabetics, which represents a large and meaningful difference. As seen in the histograms (Figure 1), the HbA1c distribution for the diabetic group was shifted to the right, reflecting poor glycemic control.

**Table 1:** Comparison of demographic and biochemical parameters between diabetic cases and non-diabetic controls. Values are mean ± standard deviation (SD). Between-group comparisons were performed using independent two-sample t-tests after checking assumptions of approximate normality and similar variances; two-sided p-values are reported. A lower p-value indicates stronger evidence of a difference between groups; p < 0.05 was considered statistically significant. HbA1c was markedly higher in cases than controls, indicating poorer glycemic control among diabetics. Total T3 and total T4 were significantly lower in cases, whereas TSH did not differ significantly. The lipid profile showed higher total cholesterol, triglycerides, LDL, and VLDL in the diabetic group; HDL was also higher in cases in this dataset. Units: age (years), HbA1c (%), TSH (µIU/mL), total T3 (ng/mL), total T4 (mcg/dL), total cholesterol (mg/dL), triglycerides (mg/dL), HDL (mg/dL), LDL (mg/dL), VLDL (mg/dL). SD: standard deviation; HbA1c: glycated hemoglobin; TSH: thyroid-stimulating hormone; T3: triiodothyronine; T4: thyroxine; HDL: high-density lipoprotein cholesterol; LDL: low-density lipoprotein cholesterol; VLDL: very-low-density lipoprotein cholesterol.

Parameter	Cases (Mean ± SD)	Controls (Mean ± SD)	p-value
AGE	49.28 ± 10.59	49.58 ± 18.03	0.886
HbA1C (%)	7.42 ± 1.46	4.83 ± 0.45	<0.001
TSH (μIU/ml)	2.44 ± 1.09	2.15 ± 1.05	0.06
Total T3 (ng/ml)	1.02 ± 0.25	1.38 ± 0.34	<0.001
Total T4 (mcg/dl)	6.32 ± 1.77	8.60 ± 2.04	<0.001
CHOL (mg/dl)	207.34 ± 40.80	162.67 ± 23.26	<0.001
TRIG (mg/dl)	225.81 ± 79.80	97.65 ± 29.40	<0.001
HDL (mg/dl)	56.68 ± 9.81	48.33 ± 5.66	<0.001
LDL (mg/dl)	113.72 ± 20.48	75.05 ± 14.34	<0.001
VLDL	47.48 ± 13.66	23.40 ± 10.51	<0.001

**Figure 2:**
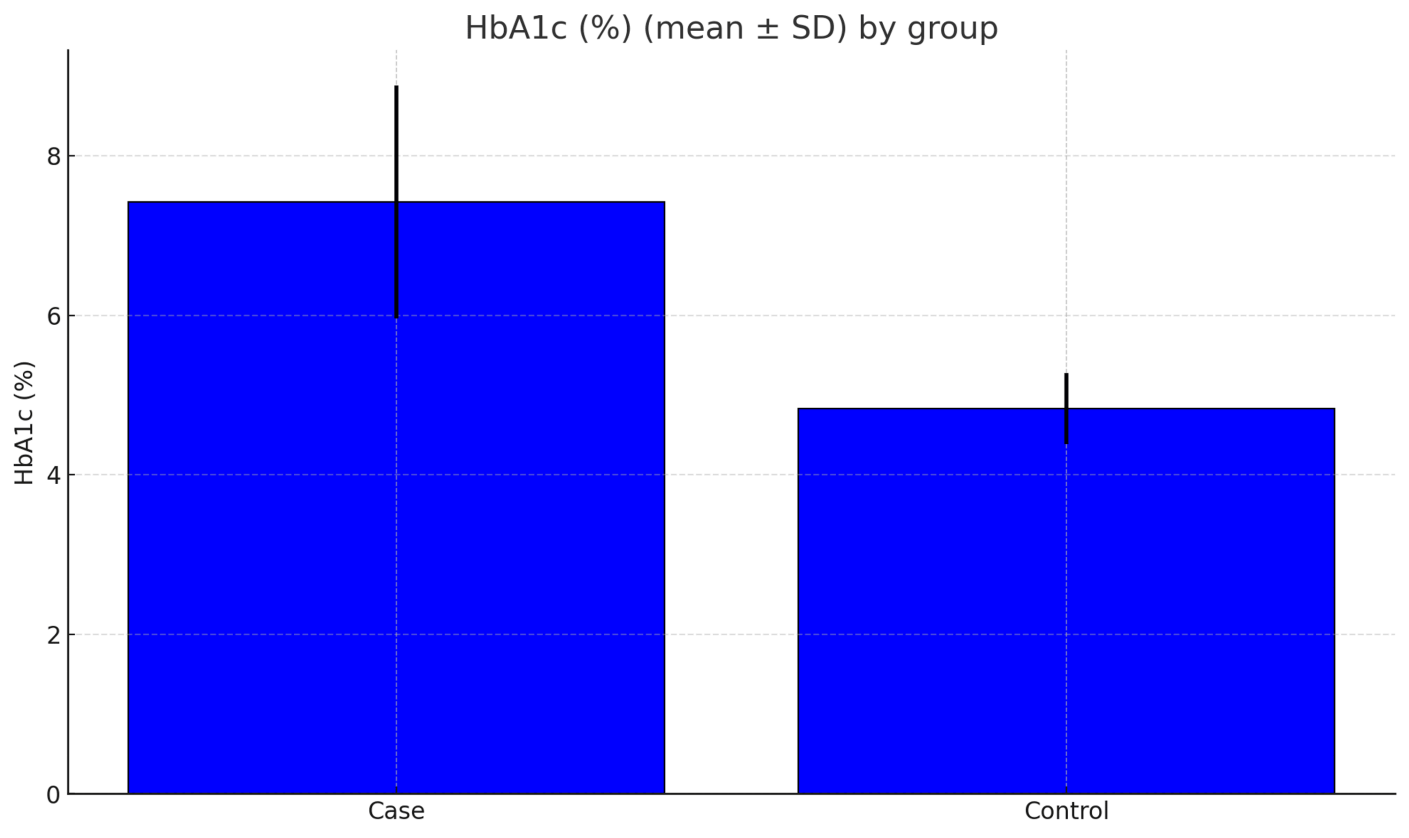
Mean glycated hemoglobin (HbA1c, %) in diabetic cases and non-diabetic controls. The bar chart shows the mean HbA1c values (± standard deviation) for both cases (n=100) and controls (n=100). Diabetic participants had markedly higher HbA1c (7.42 ± 1.46%) compared with healthy controls (4.83 ± 0.45%), reflecting significantly poorer glycemic control (p < 0.001). The mean difference of +2.59% indicates clinically relevant hyperglycemia in the case group. Error bars represent one standard deviation from the mean.

**Figure 3:**
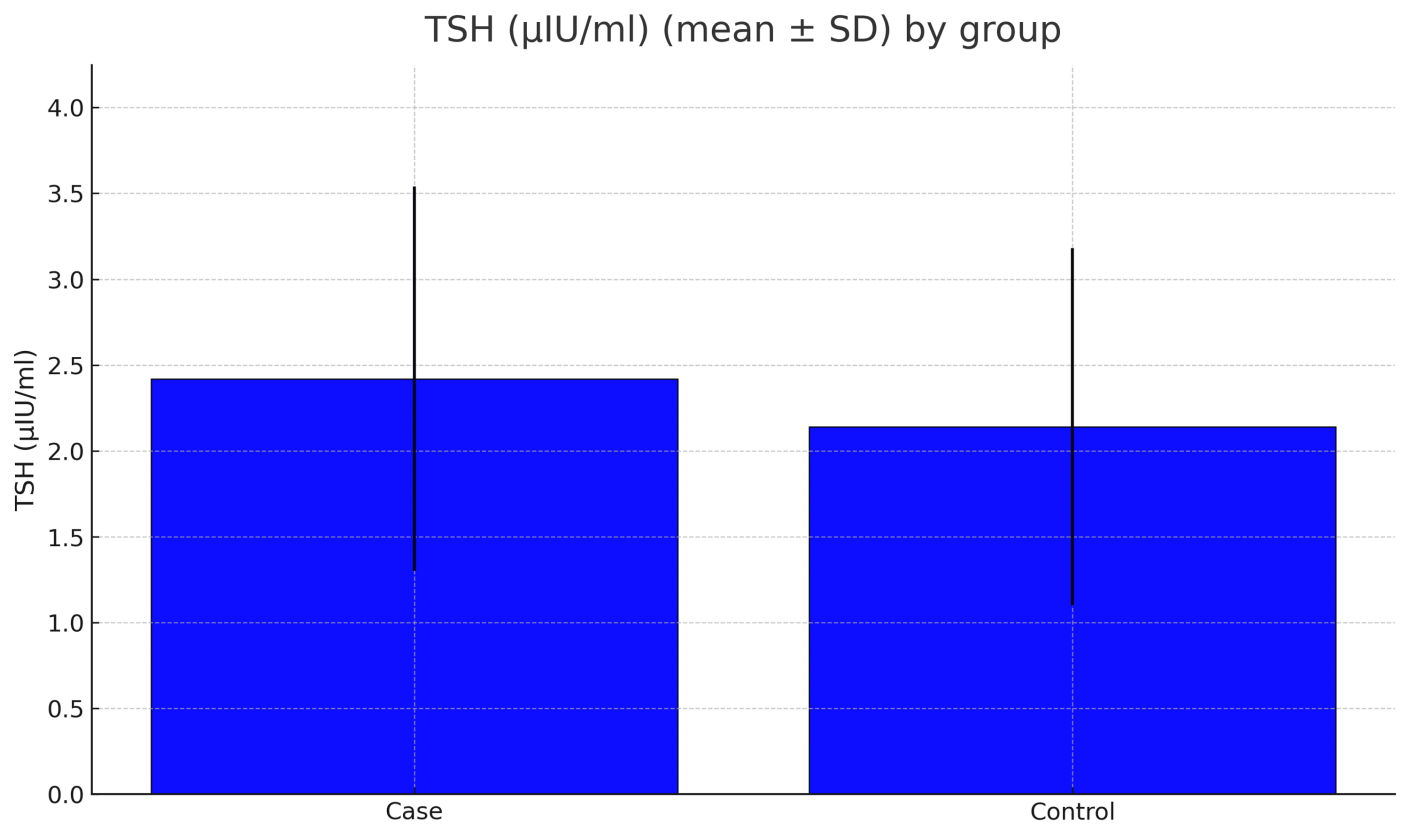
Mean thyroid-stimulating hormone (TSH, µIU/mL) in diabetic cases and non-diabetic controls. The bar chart displays the average TSH concentrations, with a standard deviation, for 100 cases and 100 controls. Cases had a mean TSH of 2.44 ± 1.09 µIU/mL, while controls showed 2.15 ± 1.05 µIU/mL. The between-group difference was not statistically significant (p = 0.060). Error bars represent one standard deviation above and below the mean, demonstrating the considerable variability of TSH levels in both groups.

The bar chart (Figure 2) highlights this again, showing that most diabetics had HbA1c values above the normal range. In the case of thyroid function tests, the average TSH was slightly higher in the diabetic group (2.44 ± 1.09 vs. 2.15 ± 1.05 µIU/mL), but the difference was not statistically significant (p = 0.060; Figure 3), but in the case of total T3 and T4 levels, they were clearly lower in diabetics compared with controls (total T3, 1.02 ± 0.25 vs. 1.38 ± 0.34 ng/mL; total T4, 6.32 ± 1.77 vs. 8.60 ± 2.04 μg/dL; both p < 0.001). This shows a consistent downward trend in these thyroid hormones among diabetics.

**Figure 4:**
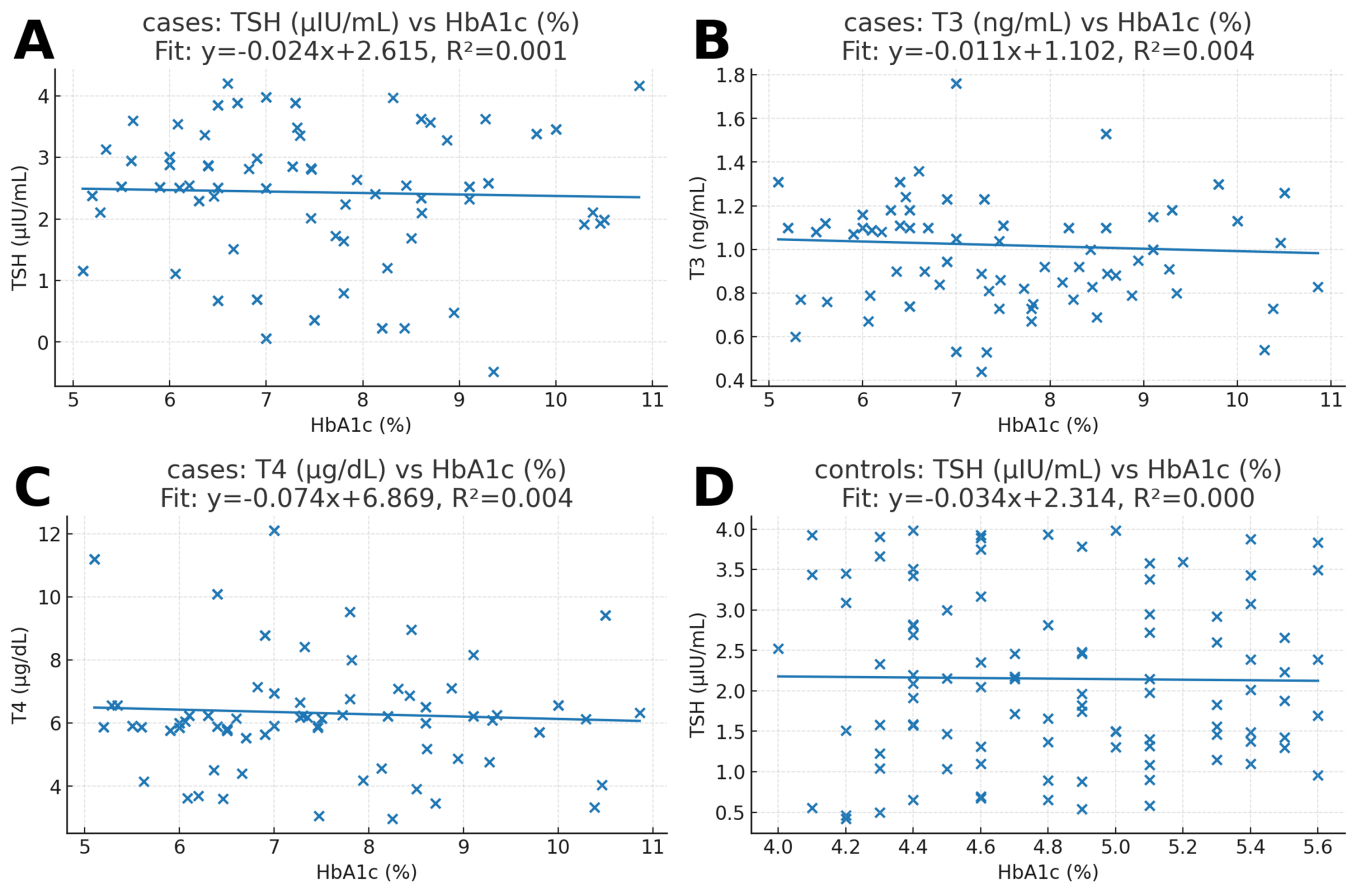
Scatterplots of HbA1c (%) versus thyroid indices in diabetic cases and non-diabetic controls. The picture above shows scatterplots that show how glycated hemoglobin (HbA1c%) and thyroid parameters are related. Panel A depicts the correlation between TSH (µIU/mL) and HbA1c in diabetic subjects (n=100). The regression line is nearly horizontal (ŷ = −0.024× + 2.615; R² = 0.001), showing a lack of significant association. Panel B shows the relationship between HbA1c and T3 (ng/mL) in cases. The slope is again weakly negative (ŷ = −0.011× + 1.102; R² = 0.004), which means that changes in HbA1c didn't have much of an effect on total T3 levels. Panel C shows how HbA1c and total T4 (µg/dL) are related in instances. It shows a slight downward tendency (ŷ = −0.074× + 6.869; R² = 0.004), but it doesn't explain much. Panel D shows the relationship between HbA1c and TSH in controls (n=100). The regression line here similarly indicated no link (ŷ = −0.034× + 2.314; R² ≈ 0.000). These scatterplots show that there was not a strong link between HbA1c levels and TSH, total T3, or total T4 concentrations in either the patients or the controls.

The scatter plots (Figure 4) were constructed to explain whether HbA1c levels are linked to thyroid parameters. In diabetics, the regression lines for HbA1c against TSH, total T3, and T4 were almost flat, and the R² values were very close to zero (0.001-0.004). This means that thyroid hormone levels did not change meaningfully with different levels of HbA1c. Similar findings were observed in controls, where HbA1c did not appear to influence TSH values (R² ≈ 0.000).

The lipid profile analysis showed significant differences (Table 1). Diabetic cases had much higher levels of total cholesterol, triglycerides, LDL, and VLDL compared with controls (all p < 0.001). Interestingly, HDL was also higher in diabetics (56.68 ± 9.81 vs. 48.33 ± 5.66 mg/dL; p < 0.001). These findings demonstrate that diabetics in this study had marked disturbances in lipid metabolism.

Overall, the study found that diabetic patients had markedly higher HbA1c levels, with lower total T3 and total T4 values and significant lipid abnormalities compared with healthy controls, and no significant correlation was observed between HbA1c and thyroid function tests in either group.

## Discussion

Key findings

In this case-control study (n = 200), the diabetes group had higher HbA1c than controls, confirming poorer glycemic control. TSH did not differ between groups, while total T3 and total T4 were lower in cases. Lipids (total cholesterol, triglycerides, LDL, and VLDL) were higher in cases; HDL was also higher. Despite group differences, scatterplots showed weak individual-level linear associations between HbA1c and thyroid indices.

Comparison with prior work

Lower peripheral thyroid hormones with little change in TSH are consistent with reports of altered thyroid economy in metabolic illness, including the low‑total T3 pattern, and with data on thyroid disease burden among people with diabetes [7-10]. Prior studies also link thyroid dysfunction with higher HbA1c and complications in diabetes [3, 8]. Our lipid pattern accords with the literature on diabetic dyslipidemia and insulin resistance, including overproduction of VLDL and guideline emphasis on aggressive lipid management in diabetes [11-14]. The higher HDL in our cases is less typical; modest HDL increases can occur with lipid‑modifying pharmacotherapy such as statins or fibrates, which may partially explain this signal in observational data [15, 16].

Biological rationale

Thyroid and insulin pathways intersect at several points. Thyroid hormone modulates glucose transport in skeletal muscle, including effects on GLUT4 expression and translocation, which may influence insulin sensitivity [4, 17, 18]. Diabetes‑related iron and oxidative stress pathways may also play roles: higher ferritin is associated with insulin resistance and incident type 2 diabetes across cohorts and meta‑analyses [5,19,20]. These mechanisms align with our observation of lower total T3/T4 and adverse lipid profiles in the case group.

Clinical implications

Together, higher HbA1c, lower total T3/T4, and adverse lipids support routine thyroid assessment and comprehensive lipid profiling in adults with diabetes, particularly when glycemic targets are not met. Given weak point‑wise correlations between HbA1c and thyroid measures, HbA1c alone should not guide thyroid testing; targeted screening remains appropriate. These clinical implications are hypothesis-generating - thyroid assessment may be considered in selected contexts but is not supported as routine solely on the basis of glycemic status.

Strengths and limitations

This present case-control study benefited from age- and sex-matched controls and standardized laboratory assays with routine internal quality control, which supported measurement reliability. But because of the single-center setting, modest sample size, and absence of several important covariates (e.g., BMI, diabetes duration, medication use, and autoimmune status), generalizability is limited and causal inference is precluded, and the unexpectedly higher HDL in cases may reflect use of lipid-modifying therapies (e.g., statins/fibrates); because medication data were not collected systematically, this observation warrants cautious interpretation and so is discussed in the Limitations. A post-hoc power check indicated that with n=100 per group (two-sided α=0.05), the study was adequately powered to detect large between-group differences - consistent with the large HbA1c effect observed but smaller effects and correlations may have been missed.

Future research

Prospective studies with detailed phenotyping (anthropometry, autoantibodies, medications, and lifestyle), iron and inflammatory indices, and multivariable adjustment are needed. Longitudinal follow‑up across HbA1c strata could clarify when thyroid changes become clinically meaningful and whether they modify cardiometabolic risk.

## Conclusions

In this case-control study, diabetic patients have significantly higher HbA1c values compared to healthy controls, confirming less glycemic control. The cases also show lower T3 and T4 levels, while TSH did not differ significantly, suggesting subtle alterations in thyroid function associated with diabetes. Cases had markedly higher lipid levels, including total cholesterol, triglycerides, LDL, and VLDL, indicating a relationship between dyslipidemia and Scatterplot analyses showed negligible correlations between HbA1c and thyroid indices, confirming that thyroid hormone changes were not linearly explained by glycemic control in this cohort.

Our study was limited by its single-center design, modest sample size, and six-month duration. The findings provide valuable insight into the biochemical interactions between diabetes and thyroid function. Clinically, the results emphasize the importance of routine biochemical monitoring, including thyroid hormone and lipid assessments, in diabetic patients to screen for early detection of metabolic abnormalities. Future multicentric studies with larger cohorts are required to confirm these associations and explore whether early thyroid screening and intervention can improve metabolic and cardiovascular outcomes in patients with diabetes.
